# 

**Published:** 1894-03

**Authors:** 


### NOTICES.

### Illinois State Dental Society.

The thirtieth annual meeting of the Illinois State Dental Society will be held in the Senate Chamber, Springfield, Ill., May 8, 9, 10 and 11, 1894. An interesting program is in the course of preparation. Practitioners of Illinois and of adjoining States are cordially invited to attend.

LOUIS OTTOFY, Secretary.

### Notice.—Alabama Dental Association.

DECATUR, ALA., JANUARY 15, 1894.

*Dear Doctor:*—Owing to the burning of one of the hotels in Troy, the Executive Committee of the Alabama Dental Association have decided that it is best to change the place of meeting from Troy to Montgomery. Meeting to be held from 10th to 13th of April, inclusive. Everything is being done to make it an interesting occasion. You are most earnestly requested to be present. Yours fraternally,

S. W. FOSTER, Secretary.

### American Medical Association.

The Association Train will leave Chicago Monday, May 28, via Santa Fe R. R., Denver & Western, and Southern Pacific, for San Francisco via Denver, Colorado Springs, Leadville, Manitou, Glenwood Springs, Salt Lake, Ogden, Truckee and Sacramento. Returning, after the meeting, the train will pass through Sacramento and Northern California to Portland, thence east by way of the Northern Pacific R. R. to St. Paul. A stop-over at Yellowstone National Park for those who desire it has been arranged, and it is understood that at several places on the journey there will be short stops. President Hibberd's party in a special car join the train at Chicago, and the St. Louis party are expected to join at Kansas City. From all points east and south, concentration on this train should be effected at Chicago and St. Louis.

### THE DENTAL REGISTER,

A MONTHLY MAGAZINE DEVOTED TO THE INTERESTS OF THE

**DENTAL PROFESSION.**

*Terms Reduced to* $2.00 *Per Year. Single Copies,* 25 *Cents.*

**EDITED BY PROF. J. TAFT, D.D.S.**

—AND—

**Published by SAM'L A. CROCKER & CO., Ohio Dental and Surgical Depot.**

The volume commences in January and closes in December.

The REGISTER being issued monthly is always fresh, therefore Indispensable to the practitioner who desires to keep posted up with the new inventions and improvements which are daily developed. Neither expense nor effort will be spared to give value and variety to its contents. Articles will be illustrated with well executed engravings whenever they will add to the interest or assist to explanation.

Its extensive circulation and frequency of issue make it one of the BEST DENTAL ADVERTISING MEDIUMS in the United States.

All subscriptions must begin with January or July.

Local or special notices, five lines or less, will be inserted for $3 each insertion; over five lines, 50 cts. per line.

All advertising bills payable in advance, or at furthest, after the issue of the first number containing the advertisement. All transient advertisements to be paid for in advance.

☞CONTRIBUTIONS SOLICITED.

Exclusively Editorial Communications should be addressed to the Editor.

The latest number of the REGISTER may be ordered with or without other good at any time, and all letters should be addressed to

